# Effect of PET Image Reconstruction Techniques on Unexpected Aorta Uptake

**DOI:** 10.4274/mirt.galenos.2018.88528

**Published:** 2019-03-19

**Authors:** Hassan Hirji, Keith Sullivan, Imran Lasker, Mhd S. Sharif, Andre Nunes, Chris Shepherd, Wai-lup Wong, Bal Sanghera

**Affiliations:** 1 Northwick Park Hospital, Department of Rheumatology, London, England; 2 University of Hertfordshire, London, England; 3 Paul Strickland Scanner Centre, Mount Vernon Hospital, London, England; 4 University of East London, London, England

**Keywords:** Positron emission tomography, computed tomography, aorta, blood pool, quantitative, qualitative, analysis

## Abstract

**Objectives:**

To determine if unexpected aorta uptake seen in some patients is influenced by popular modern reconstruction algorithms using semi-quantitative and qualitative analysis.

**Methods:**

Twenty-five consecutive patients without suspected vascular disease were selected for 18F-FDG positron emission tomography/ computed tomography (PET/CT) scanning and images of the aorta were created using iterative reconstruction (IT), IT + time of flight (TOF), IT + TOF + point spread function correction (referred collectively as UHD) with and without metal artefact reduction (MAR) algorithms. An experienced radiologist created aorta and blood pool (BP) regions of interests then copied these to all reconstructions for accurate positioning before recording target aorta standardized-uptake-values (SUV_max_) and background BP SUV_mean_. Furthermore, target-to-background ratio (TBR_max_) was defined by aorta SUV_max_-to-BP SUV_mean_ ratio for more analysis.

**Results:**

For aorta SUV_max_ with IT, IT + TOF, UHD, UHD + MAR reconstructions the mean ± standard deviation recorded were 2.15±0.43, 2.25±0.51, 2.25±0.45 and 2.09±0.4, respectively. Values for BP SUV_mean_ were 1.61±0.31, 1.58±0.28, 1.58±0.28 and 1.47±0.25, respectively. Likewise, for TBR_max_ these were 1.35±0.19, 1.43±0.21, 1.43±0.19, 1.43±0.18, respectively. ANOVA analysis revealed no significant differences for aorta SUV_max_ (F(0.86) p=0.46), BP SUV_mean_ (F(1.22) p=0.31) or TBR_max_ (F(0.99) p=0.4). However, the qualitative visual analysis revealed significant differences between IT + TOF with UHD (p=0.02) or UHD + MAR (p=0.02).

**Conclusion:**

Reconstruction algorithm effect on aorta SUV_max_ or BP SUV_mean_ or TBR_max_ was not statistically significant. However, qualitative visual analysis showed significant differences between IT + TOF as compared with UHD or UHD + MAR reconstructions. Harmonization of techniques with a larger patient cohort is recommended in future clinical trials.

## Abstract (tr)

**Amaç:**

Yarı-kantitatif ve kalitatif analiz kullanarak bazı hastalarda görülen beklenmedik aorta tutulumunun popüler modern rekonstrüksiyon algoritmalarından etkilenip etkilenmediğini belirlemektir.

**Yöntem:**

Vasküler hastalık şüphesi olmayan 25 ardışık hasta 18F-FDG pozitron emisyon tomografi/bilgisayarlı tomografi (PET/BT) görüntüleme için seçildi ve iterative rekonstrüksiyon (IT), IT + time of flight (TOF), IT + TOF + point spread function düzeltme ile, metal artefact reduction (MAR) algoritmasıyla ve bu algoritma kullanılmaksızın, aorta görüntüleri oluşturuldu. Deneyimli bir uzman aorta ve kan havuzu ROI’lerini oluşturarak bunları hedef aort SUV_maks_ ve arka plan (BP) SUV_ortalama_ değerlerini kaydetmeden önce doğru pozisyonu sağlamak için tüm rekonstrüksiyonlara kopyaladı. Buna ek olarak, hedef-BP oranı (TBR_maks_), aorta SUV_maks-_BP SUV_ortalama_ oranı kullanılarak, ileri analiz için hesaplandı.

**Bulgular:**

Ortalama ± standart deviasyon aorta SUV_maks_ değeri IT, IT + TOF, UHD, UHD + MAR rekonstrüksiyonları ile 2,15±0,43, 2,25±0,51, 2,25±0,45 ve 2,09±0,4 olarak saptandı. BP SUV_ortalama_ için bu değerler 1,61±0,31, 1,58±0,28, 1,58±0,28 ve 1,47±0,25 idi. Benzer şekilde, TBR_maks_ için bu değerler 1,35±0,19, 1,43±0,21, 1,43±0,19, 1,43±0,18 olarak belirlendi. ANOVA analizi aorta SUV_maks_ (F(0,86) p=0,46), BP SUV_ortalama_ (F(1,22) p=0,31) veya TBR_maks_ (F(0,99) p=0,4) arasında istatistik olarak anlamlı fark saptamadı. Bununla birlikte kalitatif görsel analiz, UHD (p=0,02) ya da UHD + MAR (p=0,02) rekonstrüksiyonları ile yapılan IT + TOF arasında anlamlı farklılık ortaya koydu.

**Sonuç:**

Rekonstrüksiyon algoritmalarının aorta SUV_maks_ veya BP SUV_ortalama_ ya da TBR_maks_ üzerinde etkisi istatistiki olarak anlamlı değildi. Ancak kalitatif görsel analiz, UHD ya da UHD + MAR rekonstrüksiyonları ile yapılan IT + TOF arasında anlamlı farklılık ortaya koydu. İleride yapılacak klinik çalışmalarda daha geniş bir hasta grubu ile tekniklerin harmonizasyonu önerilir.

## Introduction

PET technological scanning innovations (1) have increased rapidly over the last decade leading to improved diagnostic imaging capability. Examples include routine clinical introduction of time-of-flight (TOF) scanning (2), point-spread-function correction (PSF) (3), metal artefact reduction (MAR) (4), gating (5), dose reduction techniques (6), application to radiotherapy treatment planning (7), continuous bed motion, digital detectors etc. (8). These have all contributed significantly to widespread adoption of PET as a popular clinical diagnostic imaging tool in the patient pathway today (9). 

A recognised caveat of introducing new advances in scanning technology is the necessity to compare images against scanners incorporating older and less sophisticated equipment. Corresponding concerns in image interpretation can arise e.g. with PET/computed tomography (CT) superseding PET only systems (10) or with new PET magnetic resonance imaging systems (11). For PET this comparison can apply equally to visual qualitative analysis and semi-quantitative analysis utilizing standardized uptake values (SUV). 

An increasing recognized challenge exists in qualitative and quantitative comparison of patient scans across PET/CT vendors and device-dependent image reconstruction algorithms. PET scanner harmonization against a standard has been widely used for SUV comparison between scanners and is commonly employed in multi-centre clinical trials to reduce bias (12) leading to more reliable and reproducible results. It has also been proposed that different reconstructions be applied for optimizing qualitative and quantitative analysis (13) with a review of modern harmonization strategies (14) to address differences described above.

Specifically, in the case of PET qualitative analysis, some clinicians have commented on unexpected apparent increased physiological uptake that simulates disease in the aorta and great vessels (15,16). The full cause of these observations is unclear and may comprise of multiple, complex factors including patient physiology and scanner hardware/software configuration. Further, this effect can be exacerbated by the introduction of modern imaging algorithms e.g. PSF modelling which has the potential to boost focal uptake. The role of ^18^F-FDG in diagnosis of vascular disease (17) may be undermined with the potential to mistake image reconstruction effects as PET false positives (18). Accordingly, introduction of new technology initially has the potential to lead to loss of confidence in reporting with potential misdiagnosis and unneeded further tests possibly leading to poor utilization of funding & resources (19). 

A thorough analysis of all factors thought to be responsible for apparent increased aorta uptake is challenging clinically and beyond the scope of this publication. In response, we investigated the effect of PET reconstruction techniques on ^18^F-FDG aorta uptake, in a clinical setting, to establish if apparent increased uptake in patients without known vascular disease is influenced by modern popular algorithms. We investigated 25 consecutive patients scanned using iterative reconstruction (IT), IT + TOF, IT + TOF + PSF referred to as UHD with and without MAR algorithms for a range of aorta and blood pool (BP) SUV. Aorta uptake target-to-background ratio (TBR), defined as TBR_max_=Aorta SUV_max_/BP SUV_mean_, is a commonly used metric for assessment of vasculitis and was also investigated. We compared differences between reconstruction algorithms in terms of semi-quantitative analysis and by qualitative visual assessment.

## Materials and Methods

Twenty-five consecutive patients were selected who underwent routine PET/CT studies at our centre. Exclusion criteria included non-^18^F-FDG scans and subjects with suspected large vessel vasculitis, aortitis or thoracic aortic grafts to minimize bias arising in vascular disease. Patients with metallic implants in the required fields of view, including pacemakers, were not included due to the potential for artefacts in attenuation correction. 

Subjects scanned with a Siemens Biograph mCT 64 slice PET/CT scanner were asked to fast for six hours prior to ^18^F-FDG injection. Blood glucose was recorded prior to injection with an upper limit of 10 mmol/dL applied. Patients were injected with 4.5 MBq/kg ^18^F-FDG and following a typical 90 minute uptake period scans were acquired for 3 min per bed. Subject weight average ± standard deviation (SD) was 77.1±19.8 kg, injected activity 355.6±90.5 MBq and age 62.7±11.3 years, respectively. The scanner was calibrated with recommended QA regimes implemented and daily QA pass before clinical use to ensure accuracy and consistency of scanning was maintained. Clinical IR algorithms consisted of 2 iterations and 21 subsets with a 5 mm smoothing filter and zoom of 1 on a 200x200 matrix yielding a 4.07x4.07x3 mm^3^ voxel size.

CT acquisition without contrast media was performed from the skull base to the proximal femora. Acquisition settings included tube potential 120 kVp, automatic current modulation, revolution time 0.5 s, collimation 16x1.2 mm, pitch 0.8 and slice thickness 3 mm. Patients were asked to breathe gently during CT and PET acquisition with CT data was used for attenuation correction and anatomical localization. 

2D regions of interest (ROI) were hand created by a clinician in the aorta using trans-axial CT slices for anatomic localization (Figure 1a). ROIs were transferred to PET UHD reconstructions and adjusted if necessary to avoid adjacent activity before application *in situ* to other reconstructions. Aorta ROI (Figure 1b), and mediastinal BP ROI (Figure 1c), were acquired at the upper part of the descending aorta just below the arch where the descending aorta has a continuous circular wall. These were delineated by the outer voxels of the aortic wall and the outermost voxels of blood within the aorta at that level, respectively. Care was taken to exclude any mediastinal lymph nodes or other avid pathology within the ROI.

Two ROIs per patient (aorta SUV_max_ and BP SUV_mean_) per image reconstruction technique applied were generated and including TBR_max_ estimation amounted to 300 measurements in total across all reconstructions and all patients. Qualitative and semi-quantitative analysis was implemented on a Siemens dedicated workstation (Syngo.via, Siemens, Erlangen, Germany).

### Semi-quantitative Analysis

For semi-quantitative comparison, ROI defined aorta SUV_max_ and BP SUV_mean_ standardized to body weight were recorded using IT, IT + TOF, UHD and UHD + MAR reconstruction algorithms. TBR_max_ derived from these SUV were then calculated.

Data were investigated using one-way analysis of variance (ANOVA) revealing any statistically significant differences between means of independent reconstruction algorithms. Fischer’s least significant difference post-hoc test was applied to identify which, if any, reconstruction algorithm means were statistically different within these respective groups.

### Qualitative Analysis

Visual comparison was made by a radiologist with 1.5 years experience of PET/CT reporting, using images reconstructed by IT + TOF as the standard as compared to more recent UHD or UHD + MAR. A scoring system, for UHD or UHD + MAR in comparison with respective IT + TOF scans, was adopted such that a score of ‘1’ depicted aorta markedly less avid, ‘2’ specified aorta slightly less avid, ‘3’ represented no discernible difference, ‘4’ indicated aorta is slightly more avid while ‘5’ signified aorta markedly more avid.

The scoring system led to a parametric preference scale from which a mean and 95% confidence interval (CI) were evaluated. A consistent preference for 1 scan in the direction indicated by the coding at a 5% level was suggested when the 95% CI did not cross 0 and was consistent with a 1-sample t-test.

This project involving comparison and quality assurance of existing techniques was classified as an audit under NHS Research and Development Guidelines 2006, and therefore NHS Research and Ethics Committee approval was not required. All scans once identified as eligible under the suitability criteria were anonymized by a technician prior to further analysis by a clinician.

## Results

### Semi-quantitative

**Figure 2:**
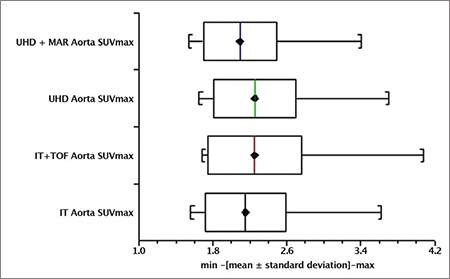
Aorta SUV_max_ distributions with different reconstructions

**Figure 3:**
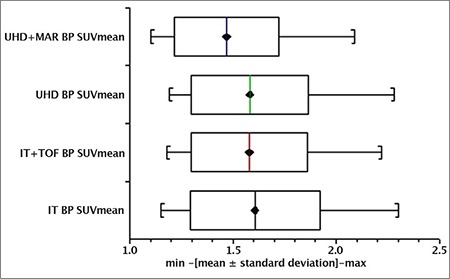
Blood pool SUV_mean_ distributions with different reconstructions

**Figure 4:**
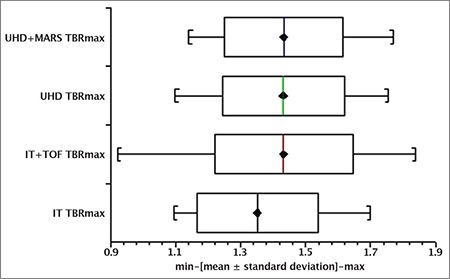
Target-to-background ratio maximum distributions with different reconstructions

A box and whisker plot (Figure 2) represented aorta SUV_max_ recorded in ROI measurements collected from the 25 patients scanned. The mean ± SD for IT, IT + TOF, UHD, UHD + MAR reconstructions was 2.15±0.43, 2.25±0.51, 2.25±0.45 and 2.09±0.4, respectively. Likewise, Figure 3 represents these parameters for BP SUV_mean_ with mean ± SD values of 1.61±0.31, 1.58±0.28, 1.58±0.28 and 1.47±0.25, respectively. Similarly, Figure 4 reveals TBR_max_ mean ± SD values of 1.35±0.19, 1.43±0.21, 1.43±0.19, 1.43±0.18, respectively.

The Shapiro-Wilkes test established non-normal behaviour in reconstruction algorithm SUV distributions necessitating log transformations for further statistical analysis. ANOVA revealed no statistically significant differences between the means of independent reconstruction algorithms investigated for aorta SUV_max_ (F(0.86) p=0.46), BP SUV_mean_ (F(1.22) p=0.31) or TBR_max_ (F(0.99) p=0.4).

### Qualitative

The appearance of standard IT + TOF reconstructions was compared with UHD or UHD + MAR algorithms and in each case the radiologist’s qualitative scoring response ranged from ‘1’ i.e. aorta markedly less avid, through to ‘5’ i.e. aorta markedly more avid yielding a score mean ± SD with associated p values of 3.28±0.58, p=0.02 or 3.29±0.59, p=0.02 for UHD or UHD + MAR, respectively, when compared with IT + TOF reconstructions.

## Discussion

Complicated automated approaches have been used elsewhere to perform segmentation typically using CT to define the aorta (20) initially. In this publication, exotic segmentation software techniques were not available while fixed uptake thresholds proved unreliable for defining aorta or BP structure accurately. Segmentation was performed manually by a trained and experienced clinician using hand drawn ROIs for delineation of relevant structures. This pragmatic approach enabled ROIs to be accurately mapped to other reconstructed scans ensuring reproducibility of placement for accurate SUV measurements.

Pre-clinical PET image reconstruction has been reported to heavily influence atherosclerotic plaque ^18^F-FDG SUV in a rabbit model (21). Clinical application of different PET reconstruction methods in oncology is known to influence SUV semi-quantification with variability introduced in SUV_max_ and SUV_mean_ (22). TBR_max_ traditionally used as a quantitative measure in vascular imaging as the ratio of vessel wall SUV_max_ to the BP SUV_mean_ is known to be a reliable index (23). As a ratio of SUVs it minimizes variability associated with patient weight, injected activity and post injection uptake times that may influence individual SUV. Therefore, TBR_max_ was also included as a metric along with individual SUVs recorded. SUV_peak_ though claimed to be more reproducible (24) is not used in widespread routine clinical practice and accordingly this publication focused on SUV_max_, SUV_mean_ and TBR_max_ indices for quantitative investigation. 

Box and whisker plots (Figures 2, 3, 4) depict minimum, maximum, mean ± SD for Aorta SUV_max_, BP SUV_mean_ and TBR_max_ with individual reconstructions, respectively. The uptake values presented in this publication are consistent with those reported elsewhere (25). In this study, no significant statistical differences were observed with different reconstruction algorithms for Aorta SUV_max_ or BP SUV_mean_ or TBR_max_ using ANOVA tests on log transformed data; suggesting that image reconstruction did not heavily influence aorta structure uptake values in our cohort of patients without known vascular disease. This result implies that unexpected enhanced uptake seen in more sensitive and accurate modern scanners is possibly related to atherosclerotic plaques not seen in earlier generation machines. The aetiology of this is not yet fully understood and may involve macrophage activity (16) warranting further investigation.

For qualitative evaluation, a trained radiologist compared IT + TOF against UHD or UHD + MAR using the scoring system described earlier. A mean value of 3.28±0.58 was scored for UHD, and 3.29±0.59 for UHD + MAR. In both cases, statistically significant differences of p=0.02 were noted confirming that UHD or UHD + MAR algorithms influenced visual assessment as compared to more traditional IT and TOF reconstruction alone. 

It is recognized that there can be a disparity of results in publications dealing with aorta uptake and image interpretation using ^18^F-FDG PET scanning, highlighting the subtlety of imaging this structure. One must also be careful to understand and interpret the effects of the image reconstruction software applied to generally diffuse aorta uptake compared with the more focal uptake typical in oncology. A systematic review article highlighting ^18^F-FDG PET uptake in patients with aortic aneurysms demonstrated conflicting results regarding prediction of aneurysm rupture and growth between studies (26). Similarly, no differences were seen in ^18^F-FDG uptake between heavily and non-heavily calcified aneurysms (27). This intricacy is also revealed in CT angiography studies where aortic signal-to-noise and contrast ratio measurements on patients reconstructed with and without Adaptive Statistical Iterative Reconstruction revealed contradictory qualitative evaluation between reviewers (28). 

Our study reflected the existing complexity reported in this field showing semi-quantitative aorta related structure uptake seen in some patients without known vascular disease is not statistically influenced by reconstruction technique. However, some caution must be exercised as our results also confirmed that new image reconstruction techniques can influence the visual appearance of aorta geometry (28), though differences were relatively small. Incongruity between quantitative and qualitative analysis has been observed in healthcare research studies and documented previously (29) supporting the findings of this study. To maintain efficacy and reduce bias from all possible sources described earlier, some form of harmonisation is recommended to ensure consistency in PET vascular imaging (12,14,26) in future investigations.

### Study Limitations

This study dealt with the consequence of manipulating various commonly used image reconstruction parameters in a clinical setting to investigate their effect on quantitative and qualitative aspects of unexpected aorta uptake in PET/CT images. The intention was not to characterize or optimize all possible parameters e.g. partial volume correction, post filter, image matrix size as this was beyond the scope of this publication. 

In terms of direct study limitations, a single radiologist created ROIs and took all measurements and performed qualitative evaluations. Ideally consensus agreement between 2 reporters would have the potential for reducing any inherent bias in results. A single image slice in each case was used to define ROIs for characterizing aorta wall, or BP and it is acknowledged that TBR values can be susceptible to partial volume effect (30) in PET scans.

However, for each patient different reconstruction techniques used in this study were applied robustly to the same ROIs on the same slice supporting accurate data acquisition and analysis with minimal additional bias. All analysis was validated by a trained and experienced statistician. We recommend a larger cohort of patients for a more detailed investigation of reconstruction parameters influencing apparent aorta ^18^F-FDG uptake in future investigations.

## Conclusions

Modern PET/CT systems can show unexpected aortic wall uptake in patients without known vascular disease. In this study, we identified that qualitative analysis revealed statistically significant differences between traditional IT + TOF reconstructions and UHD with or without MAR algorithms; indicating that image reconstruction does influence subjective image interpretation. However, quantitatively our study demonstrated little effect of reconstruction algorithm on Aorta SUV_max_, BP SUV_mean_ or TBR_max_. Consequently, a need for PET scan harmonization is recommended with a larger study cohort in future multi-centre studies.

## Figures and Tables

**Figure 1a:**
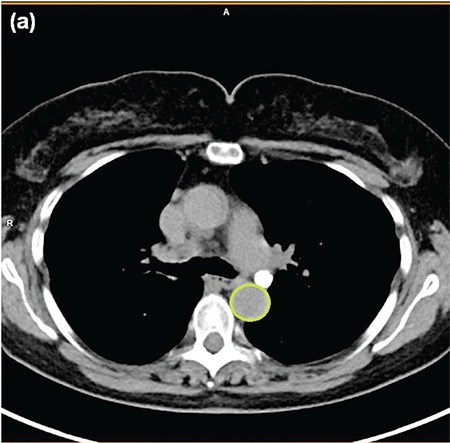
Typical regions of interest placement for the aorta guided by computed tomography

**Figure 1b:**
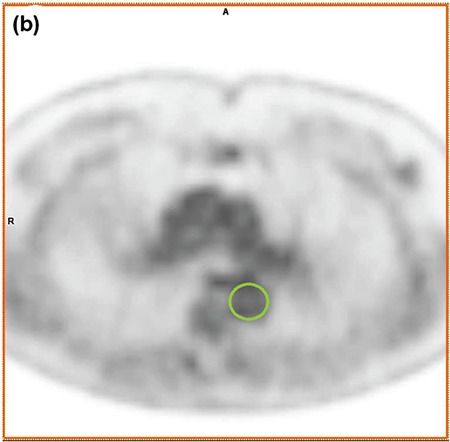
Aorta regions of interest copied to positron emission tomography slice

**Figure 1c:**
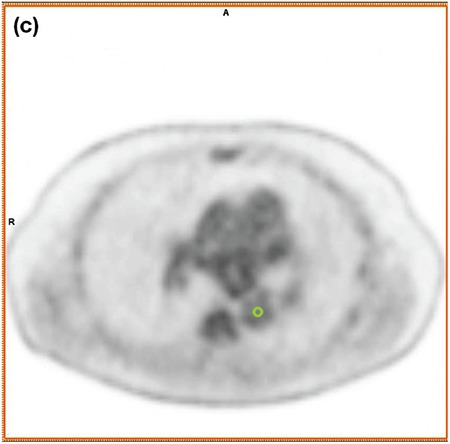
Typical positron emission tomography blood pool regions of interest
